# Ezetimibe inhibits the migration and invasion of triple‐negative breast cancer cells by targeting TGFβ2 and EMT

**DOI:** 10.1002/2211-5463.13797

**Published:** 2024-03-26

**Authors:** Lingkai Kong, Qinyu He, Ding Ma, Weiwei Shi, Qilei Xin, Chunping Jiang, Junhua Wu

**Affiliations:** ^1^ Jinan Microecological Biomedicine Shandong Laboratory China; ^2^ State Key Laboratory of Pharmaceutical Biotechnology, National Institute of Healthcare Data Science at Nanjing University, Jiangsu Key Laboratory of Molecular Medicine, Medical School Nanjing University China

**Keywords:** breast cancer, cholesterol, ezetimibe, metastasis, TGFβ2

## Abstract

The important role of cholesterol in tumor metastasis has been widely studied in recent years. Ezetimibe is currently the only selective cholesterol uptake inhibitor on the market. Here, we explored the effect of ezetimibe on breast cancer metastasis by studying its impact on breast cancer cell migration, invasion, and epithelial‐mesenchymal transition (EMT). Differential gene expression analysis and validation were also carried out to compare ezetimibe‐treated and untreated breast cancer cells. Finally, breast cancer cells overexpressing TGFβ2 were constructed, and the effect of TGFβ2 on the migration and invasion of ezetimibe‐treated breast cancer cells was examined. Our results show that ezetimibe treatment of breast cancer cells inhibited cell migration, invasion, and EMT, and it significantly suppressed the expression of TGFβ2. Overexpression of TGFβ2 reversed the inhibitory effect of ezetimibe on the migration and invasion of breast cancer cells. Taken together, our results suggest that ezetimibe might be a potential candidate for the treatment of breast cancer metastasis.

## Graphical abstract

The important role of cholesterol in tumor metastasis has been widely studied recently. Ezetimibe, the only selective cholesterol uptake inhibitor on the market, inhibits the migration and invasion of triple‐negative breast cancer cells by targeting TGFβ2 and EMT without relying on inhibiting cholesterol uptake. Overall, our findings suggest a unique mechanism by which ezetimibe prevents breast cancer metastasis.

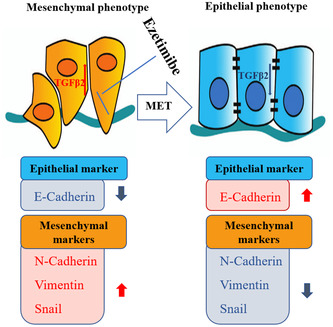

**Abbreviations**
BCA: bicinchoninic acid assay
CCK‐8: Cell Counting Kit‐8
DMSO: dimethyl sulfoxide
ECL: electrochemiluminescence
EMT: epithelial‐mesenchymal transition
FPKM: fragments per kilobase per million
HMG‐CoA: hydroxy‐3‐methylglutaryl‐coenzyme A reductase
HRP: horseradish peroxidase
IC_50_
: half maximal inhibitory concentration
MOI: multiplicity of infection
mRNA: messenger RNA
qRT‐PCR: real‐time quantitative reverse transcription PCR
RNA: ribonucleic acid
SCID mice: severe combined immune deficiency mice
TBST: tris buffered saline with tween
TGFB2: transforming growth factor beta 2

Cancer ranks second in mortality among chronic noncommunicable diseases worldwide [1]. In 2020, breast cancer had the highest mortality rate among female malignancies [2]. Various approaches have been attempted for cancer treatment, mainly divided into traditional therapy and immunotherapy [3, 4]. Current common treatments for breast cancer include surgical resection, radiotherapy, chemotherapy, and targeted therapy [5, 6, 7, 8]. However, current chemotherapeutics for breast cancer often cause drug toxicity, such as gastrointestinal adverse effects and liver and kidney function damage, while killing tumor cells [9], which brings great difficulties for clinical treatment. Targeted therapy is prone to induce drug resistance and there is currently no effective target for triple‐negative breast cancer [10]. Therefore, it is urgent to develop safe and effective drugs for breast cancer.

Tumor metastasis is one of the major causes of clinical treatment failure and recurrence in patients with breast cancer [11, 12]. Studies have shown that cholesterol can promote tumor metastasis [13, 14]. Cholesterol, a key component of cell membranes, is closely related to tumor progression [15, 16, 17]. Several studies have shown that high cholesterol can promote the growth and metastasis of lung adenocarcinoma and breast cancer [18, 19, 20, 21, 22]. In addition, high cholesterol levels are associated with breast cancer recurrence [11, 23]. These results suggest that cholesterol promotes the metastasis of breast cancer.

Currently, drugs that interfere with cholesterol metabolism through different mechanisms have been used in antitumor research [24]. Statins, the most common class of drugs that intervene in cholesterol synthesis, act by inhibiting 3‐hydroxy‐3‐methylglutaryl‐coenzyme A (HMG‐CoA) reductase, the rate‐limiting enzyme of the mevalonate pathway, and the enzyme HMG‐CoA reductase (HMGCR), which catalyzes the conversion of HMG‐CoA to MVA, and is the rate‐limiting step in the biosynthesis of cholesterol [25]. Statins affect cancer cell plasticity, increasing metastatic seeding but reducing tumor formation and metastatic growth [26]. Studies have shown that breast cancer patients receiving lipophilic statins have significantly reduced rates of recurrence [27] and mortality [28]. These results illustrate that cholesterol‐lowering drugs can inhibit not only breast cancer growth but also breast cancer metastasis.

Ezetimibe, the only selective cholesterol absorption inhibitor currently on the market, can block the exogenous absorption pathway of cholesterol [29], and its cancer‐suppressing effects have been reported. *In vivo* experiments demonstrated that ezetimibe inhibited tumor growth in prostate cancer‐bearing mice fed a high‐cholesterol diet by reducing angiogenesis [30]. Pelton *et al*. [31] found that in a breast cancer model of SCID mice, ezetimibe can alter the tumor microenvironment by inhibiting angiogenesis, thereby inhibiting the development of tumors. These studies demonstrated in different tumor models that ezetimibe inhibits tumor growth mainly by reducing angiogenesis. However, the effect of ezetimibe on tumor metastasis was not investigated.

As described earlier, cholesterol promotes breast cancer metastasis, and cholesterol‐lowering drugs, such as statins, can inhibit breast cancer metastasis and recurrence. Therefore, we were curious whether ezetimibe, also a cholesterol‐lowering drug, would have similar effects. In this study, we will observe whether ezetimibe can inhibit the migration and invasion of breast cancer cells and explore the possible mechanisms.

## Materials and methods

### Cell culture

MDA‐MB‐231 (human, female) cells were maintained in DMEM (catalog #319‐005‐CL; WISENT, Nanjing, China) with 10% fetal bovine serum (FBS) (catalog #086150035; WISENT, Nanjing, China) and penicillin (catalog #450‐201‐EL; WISENT, Nanjing, China): streptomycin solution (catalog #15140163; Gibco, New York, USA). 4T1 (mouse) cells were maintained in RPMI 1640 (catalog #350‐000‐CL; WISENT, Nanjing, China) with 10% FBS and penicillin: streptomycin solution. All cells were cultured at 37 °C in a humid incubator with 5% CO_2_.

### Preparation of ezetimibe

Ezetimibe (catalog #HY‐17376; MedChemExpress, New Jersey, USA) was added to the sterile tube and dissolved in DMSO solution to 100 mm before being stored at 4 °C for long‐term use. The storage concentration of ezetimibe is 100 mm, and the solution is diluted to the appropriate concentration when used.

### CCK‐8 assay

Cell viability was analyzed by Cell Counting Kit‐8 (CCK8; Beyotime, Shanghai, China) according to the manufacturer's protocols. The cells were seeded and cultured at a density of 5 × 10^3^/well in 100 μL of medium in 96‐well microplates (Corning, New York, USA). Cells were first treated with different concentrations of ezetimibe for 48 h. Then, 10 μL of CCK8 solution was added, and the cells were incubated for 1 h at 37 °C. The absorbance of each well was measured at OD450 nm using a multiwell plate reader (Bio‐Rad, Hercules, CA, USA).

### Wound healing assay

Wound healing assays were performed in 24‐well culture plates with the culture. A total of 4 × 10^4^ cells were seeded in every well for 24 h. The cell monolayer was scraped in a straight line to create a “scratch” with a p200 pipet tip, and the cells were washed with PBS twice and cultivated in serum‐free medium containing different concentrations of ezetimibe at 37 °C. The cells were photographed in four random fields by microscopy every 24 h.

### Transwell assays

Transwell assays were performed in 24‐well culture plates with a Transwell chamber (catalog #PIHP01250; Millipore, Darmstadt, Germany), and the upper compartment of the chamber was precoated with Matrigel (catalog #354230; Corning). A total of 8 × 10^4^ cells were suspended in 300 μL of serum‐free medium and plated in the upper compartment when 1 mL of medium with 10% FBS was added to the lower compartment. Then, the cells were treated with different concentrations of ezetimibe. After the cells were allowed to invade at 37 °C for 24 h, the Transwell membranes were fixed with 4% paraformaldehyde and stained with 0.1% crystal violet at room temperature for 1 h. The cells in the upper chamber were completely removed. The number of cells invading the membranes was calculated in four random fields under the microscope.

### Lentivirus‐mediated overexpression

Cells were inoculated overnight in 12‐well plates at a density of 5 × 10^4^ cells per well until the cells reached 30% confluence. The human TGFB2 gene (ID: 7042) was used to infect MDA‐MB‐231 cells, and 4T1 cells were infected with the murine TGFB2 gene (ID: 21808). The lentivirus was purchased from GENEChem (GENEChem, Shanghai, China). Afterward, the cells were then infected for 24 h at a multiplicity of infection (MOI) of 10 with virus dilutions for the two cell lines. After the culture medium was changed, the cell lines stably overexpressing TGFβ2 were screened by adding 2 μm puromycin. The protein expression level of TGFβ2 was detected by western blotting.

### Quantitative RT‐PCR

Cells were inoculated overnight in 12‐well plates at a density of 2 × 10^5^ cells per well and treated with different concentrations of ezetimibe for 48 h. Total RNA was extracted using TRIzol reagent (15596‐018; Life Technologies, New York, USA), and reverse transcription was performed using the Transcriptase cDNA Synthesis Kit (R323; Vazyme, Nanjing, China) according to the manufacturer's instructions. Real‐time PCR analysis was performed in an Applied Biosystems 7500 Real‐Time PCR System using ChamQ SYBR qPCR Master Mix (Q341; Vazyme, Nanjing, China) according to the manufacturer's instructions.

The human TGFB2 primers F: TGTCCCTGCTGCACTTTTGTA, R: GGTGCCATCAATACCTGCAAATC. and the Mouse TGFB2 primers F: GGTGCTCTGTGGGTACCTTG, R: GGAAGACCCTGAACTCTGCC, were synthesized by GenScript to detect TGFB2 mRNA expression.

### Western blotting analysis

Cells were lysed in NP40 buffer on ice, after which the proteins were extracted. The concentration of total protein was measured by the BCA method. Equal amounts of harvested total proteins were loaded onto sodium dodecyl sulfate‐polyacrylamide gels and submitted electrophoresis. Then, the membranes were blocked at room temperature for 2 h and incubated with primary antibodies at 4 °C overnight, followed by HRP‐conjugated secondary antibodies at room temperature for 2 h. After washing three times in TBST, protein bands were observed using an ECL chemiluminescence system (Bio‐Rad). Primary antibodies against N‐cadherin (13116, 1 : 1000; CST, Boston, USA), E‐cadherin (3195, 1 : 1000; CST, Boston, USA), vimentin (5741, 1 : 1000; CST, Boston, USA), snail (3879, 1 : 1000; CST, Boston, USA), GAPDH (MB001, 1 : 1000; Biogot Technology, Nanjing, China), and TGFβ2 (ab167655, 1 : 1000; Abcam, Cambridge, UK) were used.

### Statistical analysis

The statistical analysis was performed with graphpad prism (San Diego, USA) 8.0. The variations between the two groups were compared using the independent *t*‐test, more than two groups were compared using one‐way/two‐way ANOVA and a statistically significant difference was defined as *P* < 0.05.

## Results

### Ezetimibe inhibits the migration of triple‐negative breast cancer cells

**Fig. 1:**
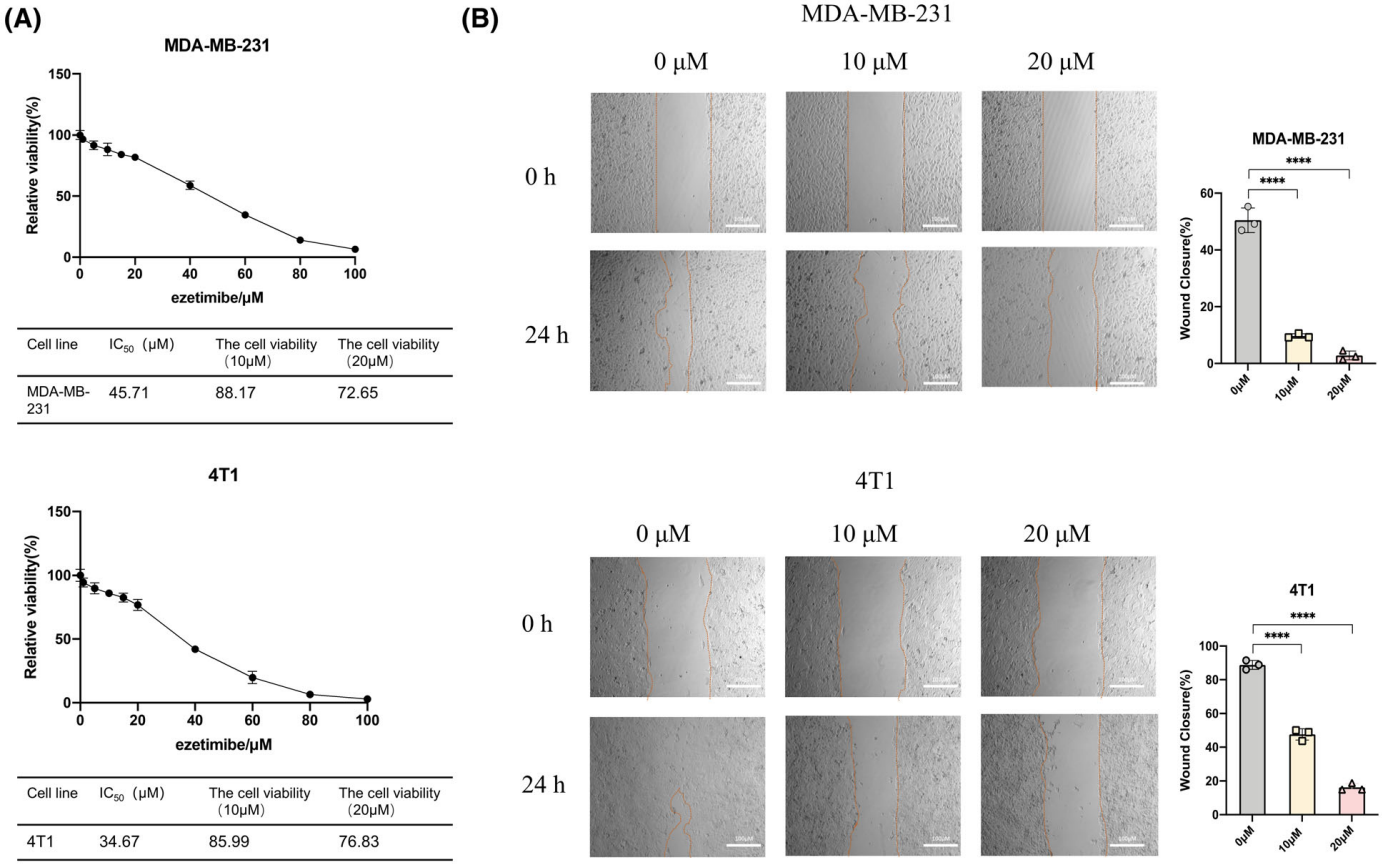
Effect of ezetimibe on the viability and migration ability of MDA‐MB‐231 and 4T1 cells. Effect of ezetimibe on the viability and migration ability of MDA‐MB‐231 and 4T1 cells. (A) MDA‐MB‐231 and 4T1 cells were treated with different concentrations of ezetimibe (0, 1, 5, 10, 15, 20, 40, 60, 80, 100 μm) for 48 h, and cell viability was assessed using the CCK‐8 assay. The experiment was repeated three times independently with six complex wells per group, and the results are shown as the mean ± standard deviation (mean ± SD). (B) MDA‐MB‐231 and 4T1 cells were treated with different concentrations of ezetimibe (0, 10, or 20 μm) for 24 h, after which a scratch healing assay was used to determine the cell healing rate. Cell images at 0 and 24 h were taken under a light microscope at a magnification of 40× in three random fields for each experimental setting. The results shown are representative of three independent experiments and are shown as the mean ± SD: ****P < 0.0001.

To determine the safe concentration of ezetimibe on MDA‐MB‐231 and 4T1 cells and evaluate its effect on the migration and invasion of these cells, we tested the effect of ezetimibe on the viability of these cells through CCK8 experiments. The viability plots of MDA‐MB‐231 and 4T1 cells after treatment with different concentrations of ezetimibe for 48 h are shown in Fig. 1A. The IC_50_ of ezetimibe on MDA‐MB‐231 and 4T1 cells for 48 h was 45.71 and 34.67 μm, respectively. After treatment with 10 μm ezetimibe for 48 h, the viability of the MDA‐MB‐231 and 4T1 cells was 88.17% and 85.99%, respectively. After treatment with 20 μm ezetimibe for 48 h, the viability of MDA‐MB‐231 and 4T1 cells was 81.83% and 76.83%, respectively. Therefore, 10 and 20 μm ezetimibe had little effect on the viability of MDA‐MB‐231 and 4T1 cells. These two concentrations were selected to study the effect of ezetimibe on the migration and invasion of MDA‐MB‐231 and 4T1 cells. Then, the wound healing assay was used to assess the effect of ezetimibe on the migration of MDA‐MB‐231 and 4T1 cells, and we found that treatment with 10 and 20 μm ezetimibe on MDA‐MB‐231 and 4T1 cells for 24 h resulted in a significant decrease in the healing rate compared with that of the untreated group, and the healing rate decreased in a dose‐dependent manner (Fig. 1B). The results indicated that ezetimibe could inhibit the migration of triple‐negative breast cancer cells.

### Ezetimibe inhibits the invasion of MDA‐MB‐231 and 4T1 cells

**Fig. 2:**
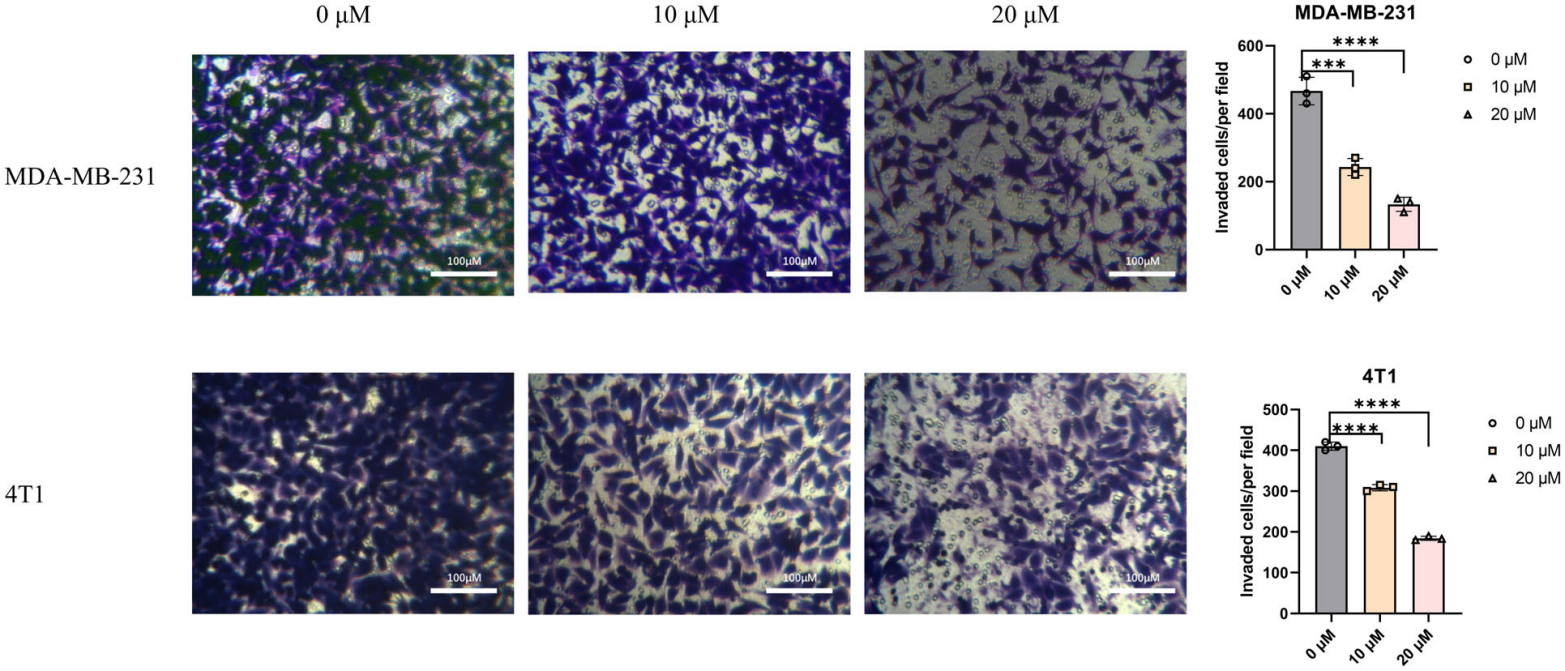
Effect of ezetimibe on the invasive ability of MDA‐MB‐231 and 4T1 cells. Effect of ezetimibe on the invasive ability of MDA‐MB‐231 and 4T1 cells. Transwell invasion assays (with Matrigel) were performed after MDA‐MB‐231 and 4T1 cells were treated with different concentrations of ezetimibe (0, 10, or 20 μm) for 24 h. Cell images at 24 h were taken under a light microscope at a magnification of 100× in three random fields for each experimental setting. The results shown are representative of three independent experiments, and the results are expressed as the mean ± standard deviation: ***P < 0.001 and ****P < 0.0001.

After observing that ezetimibe could inhibit the migration of MDA‐MB‐231 and 4T1 cells, we further explored the effect of ezetimibe on the invasion of these cells. We evaluated the effect of ezetimibe on the invasion of MDA‐MB‐231 and 4T1 cells using transwell invasion assays. As shown in Fig. 2, the numbers of MDA‐MB‐231 and 4T1 cells treated with ezetimibe for 24 h that passed through the Matrigel were significantly fewer than those of untreated cells in a dose‐dependent manner. The results illustrated that the invasive ability of MDA‐MB‐231 and 4T1 cells treated with ezetimibe was significantly reduced compared to that of untreated cells, and ezetimibe inhibited the invasion of triple‐negative breast cancer cells.

### Ezetimibe inhibits EMT in MDA‐MB‐231 and 4T1 cells

**Fig. 3:**
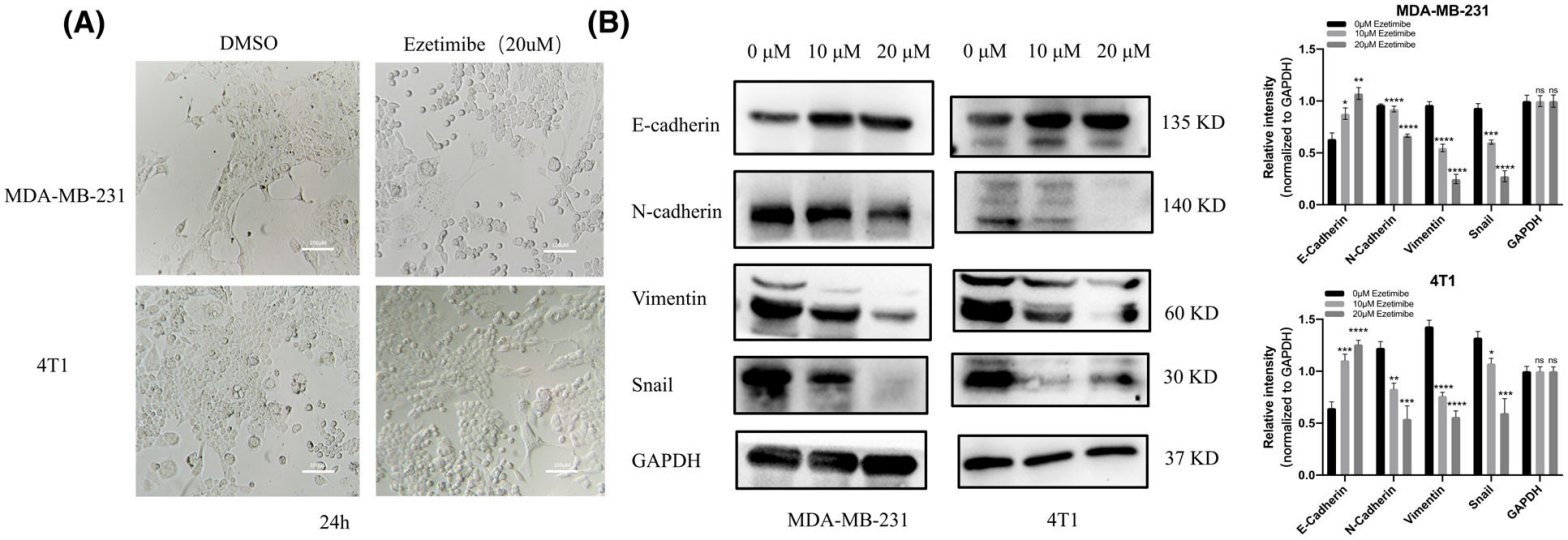
Effect of ezetimibe on the morphology and EMT‐related markers in MDA‐MB‐231 and 4T1 cells. Effect of ezetimibe on the morphology and EMT‐related markers in MDA‐MB‐231 and 4T1 cells. (A) MDA‐MB‐231 and 4T1 cells were treated with 20 μm ezetimibe for 24 h, and then cell morphology was observed and photographed by microscopy at a magnification of × 100. (B) MDA‐MB‐231 and 4T1 cells were treated with different concentrations of ezetimibe (0, 10, 20 μm) for 24 h, followed by western blot assay to detect the expression of E‐cadherin, N‐cadherin, vimentin and snail protein, and the grayscale values of these protein bands in the cells were analyzed. The results shown in the figures are representative of three independent experiments, and the results are expressed as the mean ± standard deviation: *P < 0.05, **P < 0.01, ***P < 0.001, and ****P < 0.0001.

We found that ezetimibe can inhibit the migration and invasion of MDA‐MB‐231 and 4T1 cells and that epithelial‐mesenchymal transition (EMT) plays an important role in the occurrence of invasion and subsequent metastasis of tumor cells. Therefore, we examined whether ezetimibe affects EMT in MDA‐MB‐231 and 4T1 cells. We first observed the morphology of ezetimibe‐treated MDA‐MB‐231 and 4T1 cells after 24 h by microscopy, as shown in Fig. 3A. The ezetimibe‐treated cells exhibited a cobblestone‐like shape as opposed to the spindle‐shaped DMSO‐treated control cells, with a low infiltrative and migratory competent phenotype, indicating that ezetimibe slows the morphological transition of triple‐negative breast cancer cells during EMT. We detected the expression of EMT‐related markers by western blot assay in MDA‐MB‐231 and 4T1 cells. As shown in Fig. 3B, compared with those in untreated cells, the levels of the mesenchymal markers N‐cadherin, vimentin, and snail protein were significantly downregulated, while the expression of the epithelial marker E‐cadherin was significantly increased in the ezetimibe‐treated group. These results indicate that MDA‐MB‐231 and 4T1 cells treated with ezetimibe display lower levels of EMT and that ezetimibe can significantly inhibit EMT in triple‐negative breast cancer cells, thereby affecting the migration and invasion abilities of triple‐negative breast cancer cells.

### Ezetimibe inhibits TGFβ in triple‐negative breast cancer cells

**Fig. 4:**
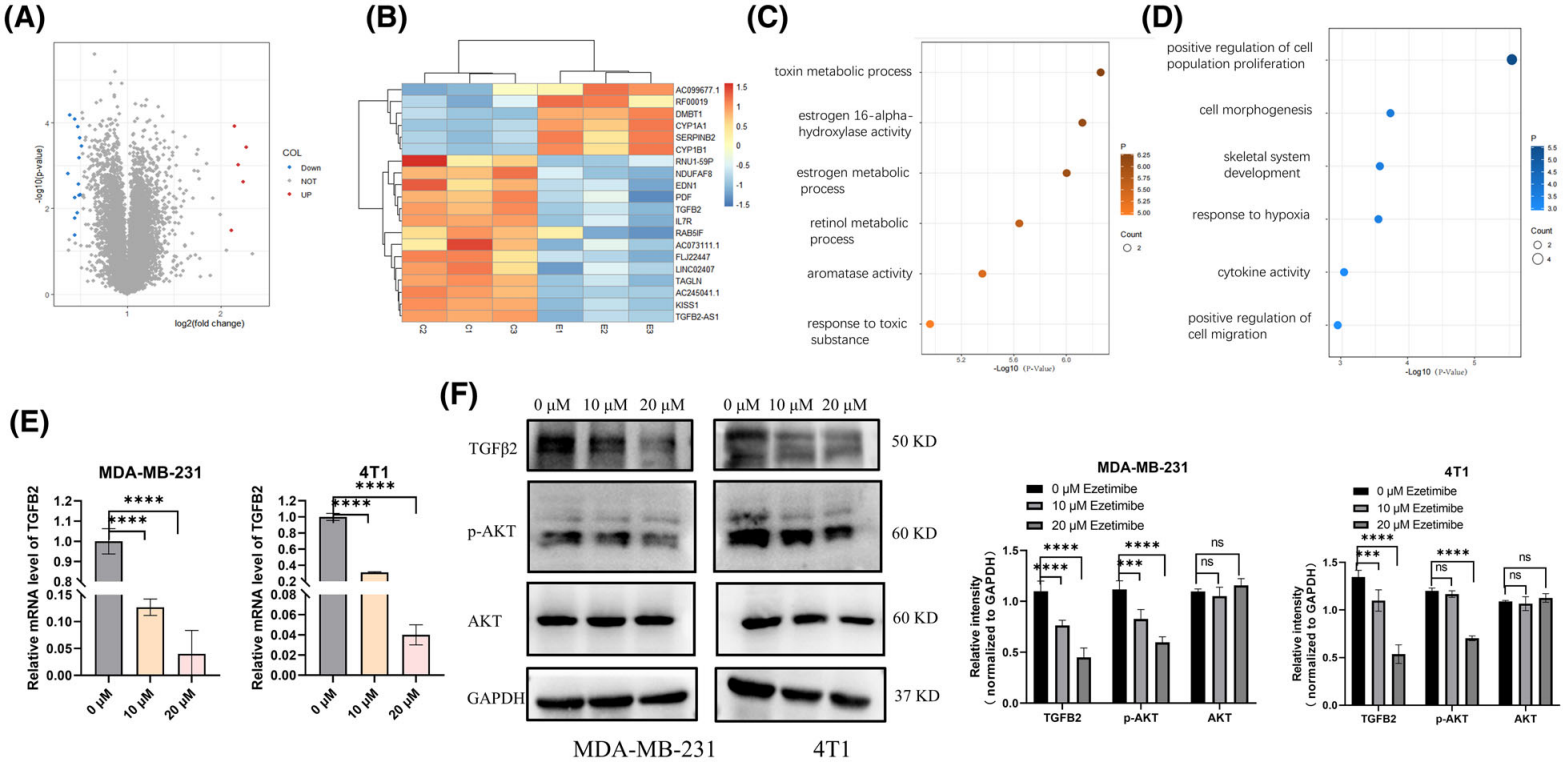
Effect of ezetimibe on the transcriptome of MDA‐MB‐231 cells and the mRNA and protein levels of TGFβ2 in MDA‐MB‐231 and 4T1 cells. Effect of ezetimibe on the transcriptome of MDA‐MB‐231 cells and the mRNA and protein levels of TGFβ2 in MDA‐MB‐231 and 4T1 cells. (A) Transcriptome sequencing was performed in MDA‐MB‐231 cells treated with 20 μm ezetimibe for 48 h. Differentially expressed genes between ezetimibe‐treated cells and control cells were then plotted as a volcano plot. (B) Differentially expressed genes between MDA‐MB‐231 cells treated with ezetimibe and control cells were selected based on the gene's fold change value using |log_2_‐fold change| ≥ 1, upregulation fold change > 2, and downregulation fold change < 0.52 as the filtering criterion. (C, D) The enriched pathways were extracted and merged based on Gene Ontology (GO) enrichment, and distribution plots were made according to the enrichment *q* value of each pathway. (C) Pathway enrichment analysis of upregulated genes in (B) is shown. (D) Pathway enrichment analysis of downregulated genes in (B) is shown. (E) qRT‐PCR experiments were performed to determine TGFβ2 mRNA in MDA‐MB‐231 and 4T1 cells after treatment with ezetimibe (0, 10, or 20 μm) for 24 h. (F) MDA‐MB‐231 and 4T1 cells were treated with different concentrations of ezetimibe (0, 10, or 20 μm) for 24 h, and then the expression of TGFβ2 was determined using western blotting, and the grayscale values of each band were analyzed according to the experimental results. The results shown are representative of three independent experiments and are expressed as the means ± standard deviations: ***P < 0.001 and ****P < 0.0001.

We have shown that ezetimibe can inhibit the migration and invasion abilities as well as EMT of triple‐negative breast cancer cells, and to define the mechanism of ezetimibe's effect, we used transcriptome sequencing to compare the differential gene expression profiles between ezetimibe‐treated and untreated MDA‐MB‐231 cells, and the gene expression profiles by transcriptome sequencing are shown in Fig. S1. We screened 146 differentially expressed genes (DEGs) based on fold change (FC) values and *P* values < 0.05. Of these, 44 genes had a difference multiple of more than 2 times, and 102 genes had a difference multiple of < 0.5. The differential gene cluster analysis plot and volcano plot of transcriptome sequencing are shown in Fig. 4A,B and Table S1. Based on the fold change values of upregulated gene ploidy > 2 and downregulated gene ploidy < 0.52, we identified six upregulated genes and 14 downregulated genes in ezetimibe‐treated cells with FPKM more significant than 1 and *P* values < 0.05. We then further subjected these DEGs to pathway enrichment analysis.

The Gene Ontology analysis results are shown in Fig. 4C,D, and according to the enrichment degree *q* value of the pathway, the upregulated gene is mainly related to the process of toxin metabolism (Fig. 4C), which may be a normal metabolic process after the treatment of cells with the cholesterol‐lowering drug ezetimibe. In particular, we observed that the expression of DMBT1 was upregulated after ezetimibe treatment. It's reported that DMBT1 can also suppress ovarian cancer proliferation, migration, and invasion [32]. At the same time, DMBT1 is also involved in protein metabolism and surfactant metabolism, which is consistent with the upregulated pathway enriched after ezetimibe treatment. It may be involved in the cell's metabolic process for ezetimibe.

Based on the enrichment degree (*q* value) of the pathways, the downregulated genes were related to cell morphology pathways, which are related to breast cancer cell migration, invasion, and EMT that we previously studied, so we focused on downregulated genes. We then filtered out two downregulated genes associated with cell morphology: il7r (interleukin 7 receptor) and tgfb2 (transforming growth factor beta 2) (Fig. 4D). Since il7r is associated with inflammation and immunity [33], by reviewing the literature to analyze the correlation between each downregulated gene and tumor migration and invasion and considering the ranking of the fold change of genes, we found that TGFβ2 in downregulated genes is closely related to tumor metastasis. TGFβ2, a member of the TGFβ family, plays a critical role in the EMT of cells [34, 35, 36]. Multiple studies have demonstrated that increased expression of TGFβ2 promotes breast cancer metastasis [37, 38]. Notably, recent studies have noted that a high‐cholesterol diet significantly induces TGFβ expression in the livers of mice [39, 40]. These results guide us to further probe the relationship between ezetimibe and TGFβ in breast cancer cells. We validated the effect of ezetimibe on TGFβ2 mRNA by qRT‐PCR, which showed a significant decrease in TGFβ2 mRNA in MDA‐MB‐231 and 4T1 cells treated with ezetimibe compared to that in untreated cells (Fig. 4E). We next tested the effect of ezetimibe on TGFβ2 protein expression by western blot assay. The expression levels of TGFβ in MDA‐MB‐231 and 4T1 cells treated with ezetimibe were significantly decreased compared with those in untreated cells (Fig. 4F). These results demonstrate that the mRNA and protein expression levels of TGFβ were significantly decreased in MDA‐MB‐231 and 4T1 cells treated with ezetimibe. We further used western blotting to detect the downstream PI3K/AKT pathway of TGFB2 in ezetimibe‐treated cells. It was found that ezetimibe significantly inhibited AKT phosphorylation and had no effect on total AKT expression (Fig. 4F). These results further demonstrated that ezetimibe inhibited TGFb and downstream PI3K/AKT pathways in triple‐negative breast cancer.

### Overexpression of TGFβ2 reverses the inhibitory effect of ezetimibe on the migration and invasion of MDA‐MB‐231 and 4T1 cells

Sequencing results and validation experiments implicated the role of TGFβ2 in the ezetimibe‐mediated inhibition of migration and invasion in triple‐negative breast cancer cells. Given that previous studies have shown that increased TGFβ2 expression promotes breast cancer metastasis, we hypothesized that ezetimibe inhibits the migration and invasion of triple‐negative breast cancer cells through TGFβ2.

**Fig. 5:**
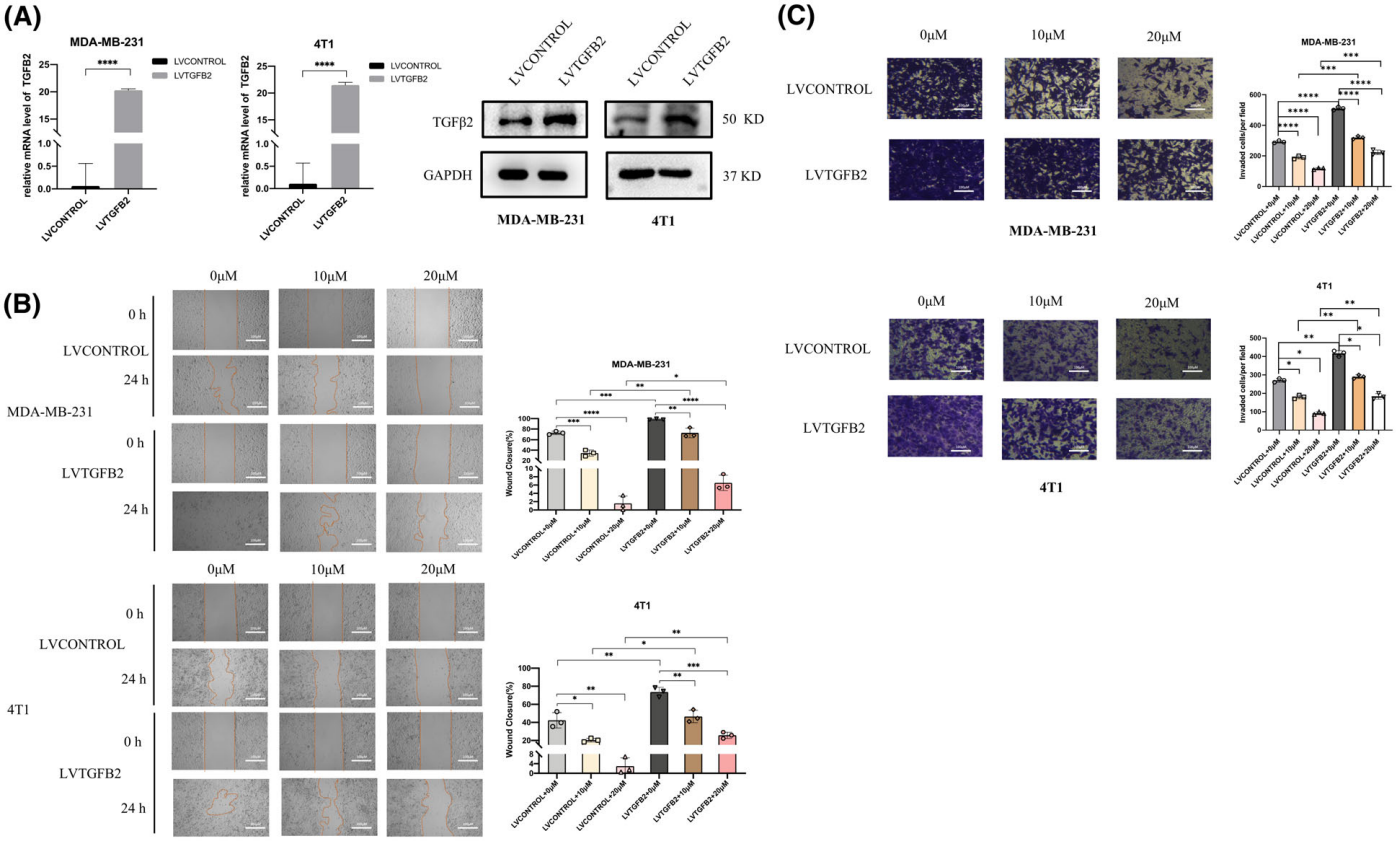
Effect of TGFβ2 overexpression on the ability of ezetimibe to inhibit cell migration and invasion in MDA‐MB‐231 and 4T1 cells. Effect of TGFβ2 overexpression on the ability of ezetimibe to inhibit cell migration and invasion in MDA‐MB‐231 and 4T1 cells. (A) Detection of TGFβ2 in TGFβ2‐overexpressing (LVTGFB2) MDA‐MB‐231 and 4T1 cells and control cells (LVCONTROL) using qRT‐PCR and western blotting. (B) TGFβ2‐overexpressing MDA‐MB‐231 and 4T1 cells and control cells were treated with different concentrations of ezetimibe (0, 10, or 20 μm) for 24 h, and then the cell healing rate was detected using a scratch healing assay. Cell images at 0 and 24 h were taken under a light microscope at a magnification of 40× in three random fields for each experimental setting. (C) TGFβ2‐overexpressing MDA‐MB‐231 and 4T1 cells and control cells were treated with different concentrations of ezetimibe (0, 10, or 20 μm) for 24 h, and then a transwell invasion assay (with Matrigel) was used to detect the number of cells that crossed the Matrigel. Cell images at 0 and 24 h were taken under a light microscope at a magnification of 100× in three random fields for each experimental setting. The results shown are representative of three independent experiments and are expressed as the means ± standard deviations: *P < 0.05, **P < 0.01, ***P < 0.001, and ****P < 0.0001.

First, we used lentiviral transfection to achieve TGFβ2 overexpression (LVTGFB2) and control cell (LVCONTROL) in MDA‐MB‐231 and 4T1 cells and verified it by real‐time PCR and western blot experiments. As shown in Fig. 5A, TGFβ2‐overexpressing MDA‐MB‐231 and 4T1 cells were constructed successfully. We next treated TGFβ‐overexpressing MDA‐MB‐231 and 4T1 cells and control cells with ezetimibe. The migration ability of the cells was assessed by a scratch assay, and the healing rate of cells overexpressing TGFβ2 was significantly higher than that of control cells (Fig. 5B), illustrating that overexpression of TGFβ2 in triple‐negative breast cancer cells resisted the inhibitory effect of ezetimibe on cell migration.

Similarly, we treated TGFβ2‐overexpressing MDA‐MB‐231 and 4T1 cells and control group cells with ezetimibe, and then the invasive ability of the cells was assessed by Transwell invasion assay. We found that the number of TGFβ2‐overexpressing MDA‐MB‐231 and 4T1 cells crossing the Matrigel was significantly higher than that of control cells (Fig. 5C), indicating that TGFβ overexpression significantly attenuated the inhibitory effect of ezetimibe on the invasion of triple‐negative breast cancer cells. Overall, we demonstrated that overexpression of TGFβ2 can reverse the inhibitory effect of ezetimibe on the migration and invasion of triple‐negative breast cancer cells. Thus, ezetimibe's ability to inhibit the migration and invasion of triple‐negative breast cancer cells occurs through TGFβ2.

## Discussion

Tumor metastasis is a significant cause of clinical therapy failure and cancer recurrence, especially in patients with breast cancer [11, 12]. Multiple studies have demonstrated a positive association between high cholesterol levels and the risk of developing breast cancer [14, 41, 42]. Targeting cholesterol metabolism may be an effective way to inhibit cancer progression [24]. For instance, ezetimibe, a drug that stops cholesterol absorption, has been found to inhibit the growth of prostate, breast, and liver cancers by inhibiting angiogenesis [30, 31, 43]. Our study showed that ezetimibe effectively inhibited triple‐negative breast cancer cell migration, invasion, and EMT. Additionally, we discovered that ezetimibe significantly decreased TGFβ2 mRNA and protein levels. This finding implies that TGFβ2 may be a crucial factor in preventing breast cancer metastasis by ezetimibe. We further observed that overexpressing TGFβ2 in triple‐negative breast cancer cells counteracted the inhibitory effects of ezetimibe on tumor cell invasion and metastasis. Our results suggest that TGFβ2 plays a critical role in metastasis and that ezetimibe prevents the metastasis of triple‐negative breast cancer by suppressing the expression of TGFβ2.

Regarding the effect of ezetimibe on negative breast cancer cells, Pelton *et al*. [31] found that inhibiting angiogenesis in triple‐negative breast cancer with ezetimibe could block the promotion of tumor metastasis caused by a high‐cholesterol diet. This implies that high cholesterol concentrations are closely related to tumor vascularization and that cholesterol‐lowering drugs could help prevent tumor metastasis by inhibiting tumor vascularization. However, our study discovered a novel mechanism for inhibiting tumor metastasis with ezetimibe: inhibiting the migration and invasion of triple‐negative breast cancer cells by suppressing TGFβ2 expression. These findings suggest that ezetimibe might inhibit tumor metastasis independently of its cholesterol‐lowering function. To demonstrate this, we treated the tumor cells with ezetimibe and then collected the supernatant for cholesterol content testing. The results showed that ezetimibe did not affect the absorption of cholesterol by the cells at the concentration of 10 and 20 μm. However, the use of lovastatin, a cholesterol synthesis inhibitor, as a control similarly hinders the migration ability of triple‐negative breast cancer cells in Fig. S2a,b. Moreover, it has been reported that cholesterol‐inhibiting drugs such as lovastatin and simvastatin can inhibit the metastasis of Triple‐Negative Breast Cancer, through dysregulation of cytoskeleton‐associated proteins [44] and FOXO3a [45]. It can be observed that the mechanism by which cholesterol‐lowering drugs inhibit the metastasis of triple‐negative breast cancer is complex. Although we have proven that ezetimibe does not affect the absorption of cholesterol by cells at concentrations of 10 and 20 μm, more comprehensive experimental verification is needed to completely rule out the influence of cholesterol‐related mechanisms.

In recent years, there has been some progress in the study of drugs targeting cholesterol metabolism in breast cancer metastasis [24]. In addition, Beckwitt *et al*. [46] found that atorvastatin at a concentration of 20 μm inhibited migration by significantly inhibiting mesenchymal breast cancer cell proliferation. While the drug used in our study was ezetimibe, which directly inhibits cholesterol absorption, and ezetimibe did not affect breast cancer cell survival, the results indicated that ezetimibe inhibits the cholesterol absorption pathway of tumor cells and can directly inhibit EMT, migration, and invasion abilities of breast cancer cells. We speculate that breast cancer cell migration and invasion may be more dependent on exogenous cholesterol uptake and that directly inhibiting this pathway might play a more significant role in inhibiting these abilities.

In addition to statins, which inhibit cholesterol synthesis, and ezetimibe, which inhibits cholesterol absorption, inhibition of exogenous lipid uptake by inhibiting proprotein convertase subtilisin/kexin type‐9 (PCSK9) is also a common strategy to lower cholesterol [47]. Studies have demonstrated that overexpression of PCSK9 inhibited EMT in colorectal cancer cells to promote tumor metastasis [48]. The ezetimibe used in this study can bind with high efficiency to the cholesterol carrier protein NPC1L1 and lower cholesterol in the blood by inhibiting the uptake of exogenous cholesterol. The results illustrate that inhibition of cholesterol synthesis or absorption pathways can effectively inhibit tumor metastasis.

Numerous studies have indicated that TGFβ2 facilitates breast cancer metastasis by promoting EMT and lipid storage in tumor cells [37, 38, 49]. To investigate how ezetimibe can impede TGFβ2 expression in breast cancer cells, we conducted RNA‐Seq experiments on drug‐treated cells. Our results showed that ezetimibe inhibits TGFβ2 expression in triple‐negative breast cancer cells. However, it has been reported that TGFB2‐AS1, as an lncRNA, can negatively regulate TGFb2 and inhibit triple‐negative breast cancer progression [38]. This seems to contradict our findings. However, our study focused on the impact of externally administered drugs on cells, and the effects on cells at varying drug concentrations are intricate and may disturb the internal homeostatic environment of the cells. Previous studies have established that agents inhibiting the TGFβ2 pathway can impede tumor metastasis [50, 51, 52]. For instance, ITD‐1, a drug that suppresses TGFβ2, prevents Smad2/3 phosphorylation, thereby inhibiting glioma cell and gastric cancer cell invasion [50, 51]. Similarly, our data revealed that ezetimibe restrains breast cancer cell migration and invasion, conceivably by targeting TGFβ2 and its downstream target PI3K/AKT pathway. To verify whether ezetimibe has a specific effect on triple‐negative breast cancer, we also assessed its inhibitory effect on metastasis in the luminal A (MCF7) and HER2‐enriched (MDA‐MB‐453) breast cancer cell lines in Fig. S2c,d. The results showed that ezetimibe did not affect the migratory ability of these two cell lines. Based on the above results, ezetimibe may be specific for inhibiting the metastasis of triple‐negative breast cancer. Our study showed that ezetimibe can inhibit the expression of TGFB2, thereby preventing cell metastasis. Cancer Cell Line Encyclopedia (CCLE) database analysis revealed that the mRNA expression of TGFB2 in MCF7 and MDA‐MB‐453 cells was lower than in MDA‐MB‐231 (Fig. S3). This suggests that ezetimibe specifically targets triple‐negative breast cancer by inhibiting TGFB2 expression and impairing EMT function.

Another mechanism by which TGFβ2 triggers tumor metastasis is by promoting angiogenesis [53]. Studies have found that inhibitors of TGFβ2, such as Trabedersen, can obstruct TGFβ2's interaction with angiogenesis‐promoting factors to halt pancreatic tumor growth and angiogenesis [52]. Moreover, Pelton *et al*. [31] demonstrated that ezetimibe stabilizes the vascular structure in a breast cancer mouse model by increasing pericyte coverage on vessels, thereby inhibiting angiogenesis and consolidating the tumor vasculature. These observations led us to hypothesize that ezetimibe restrains breast cancer cell migration and invasion by nullifying the angiogenic consequences of TGFβ2 and increasing perivascular cell coverage by trimming cholesterol absorption levels. Our findings about the novel potential effects of ezetimibe on tumor angiogenesis warrant further study.

In conclusion, this study demonstrates that ezetimibe inhibits breast cancer cell migration, invasion, and EMT and identifies TGFβ2's key role in this process. For the first time, this study reveals the effects of ezetimibe on tumor cell migration and invasion, and the underlying mechanism is the inhibition of TGFβ and EMT in cancer cells. This study provides new evidence for the regulation of cholesterol metabolism in tumor cells, which can inhibit their migration and invasion. Additionally, it presents new evidence for the use of drugs that regulate cholesterol metabolism to control the metastasis and recurrence of tumors. Furthermore, this study offers a basis for the clinical translation of ezetimibe for the treatment of breast cancer metastasis and recurrence.

## Conflict of interest

The authors declare no conflict of interest.

## Author contributions

JW and CJ provided the conceptualization, review, and editing. LK and QH investigated and performed the experiments, wrote the original draft. DM wrote the original draft. WS and QX visualized and presented the data. All authors agree to be accountable for all aspects of work ensuring integrity and accuracy.

## Supporting information


**Fig. S1.** Effect of ezetimibe on the gene expression in MDA‐MB‐231 cells.
**Fig. S2.** The effect of ezetimibe on cholesterol absorption in TNBC and its effect on cell viability and migration in luminal A (MCF7) and HER2‐enriched (MDA‐MB‐453) breast cancer cells.
**Fig. S3.** The Cancer Cell Line Encyclopedia (CCLE) database was used to analyze the mRNA expression of TGFB2 in the MCF7, MDA‐MB‐453, and MDA‐MB‐231 cell lines.
**Table S1.** Differential genes screened by an FPKM greater than 1, and p value less than 0.05, upregulated gene ploidy greater than 2 and downregulated gene ploidy less than 0.52.

## Data Availability

All data generated or analyzed during this study are included in this published article.
