# Supplementary material for: Ezetimibe inhibits the migration and invasion of triple‐negative breast cancer cells by targeting TGFβ2 and EMT
Source: FEBS Open Bio. 2024 Mar 26;14(5):831–42. doi: 10.1002/2211-5463.13797 (PMC11073500; doi:10.1002/2211-5463.13797)
Supplement: Supplementary file 1 — Fig. S1. Effect of ezetimibe on the gene expression in MDA‐MB‐231 cells. Fig. S2. The effect of ezetimibe on cholesterol absorption in TNBC and its effect on cell viability and migration in luminal A (MCF7) and HER2‐enriched (MDA‐MB‐453) breast cancer cells. Fig. S3. The Cancer Cell Line Encyclopedia (CCLE) database was used to analyze the mRNA expression of TGFB2 in the MCF7, MDA‐MB‐453, and MDA‐MB‐231 cell lines. Table S1. Differential genes screened by an FPKM greater than 1, and p value less than 0.05, upregulated gene ploidy greater than 2 and downregulated gene ploidy less than 0.52. [file FEB4-14-831-s001.docx]

**Supplementary files**


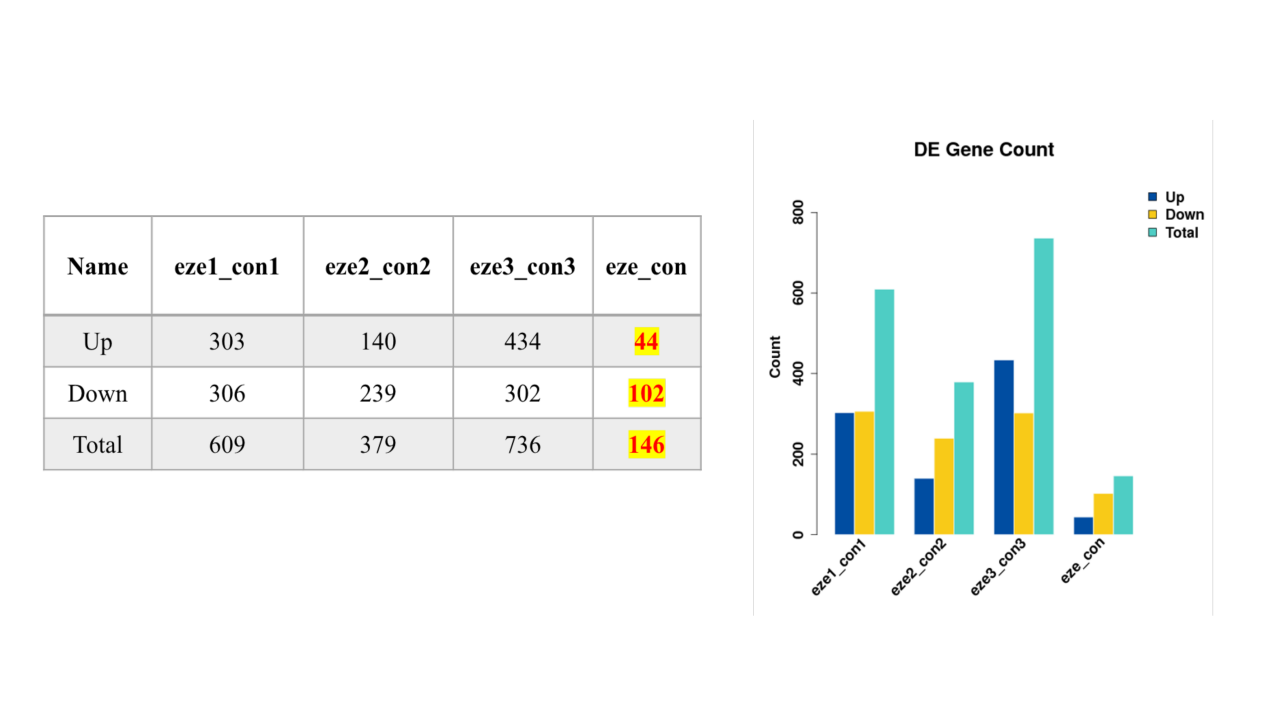


**Fig S1. Effect of ezetimibe on the gene expression in MDA-MB-231 cells.** The number of differentially expressed genes obtained by transcriptome sequencing after 24 h of ezetimibe treatment in MDA-MB-231 cells.

**Table S1 Differential genes screened by an FPKM** **greater than 1, a p value** **less than 0.05, up-regulated gene ploidy greater than 2 and down-regulated gene ploidy less than 0.52.** Based on the criteria of a fold change in upregulated genes greater than 2 and a fold change in downregulated genes less than 0.52, 6 upregulated genes and 14 downregulated genes were identified in exemestane-treated cells with a FPKM greater than 1 and a P value less than 0.05.


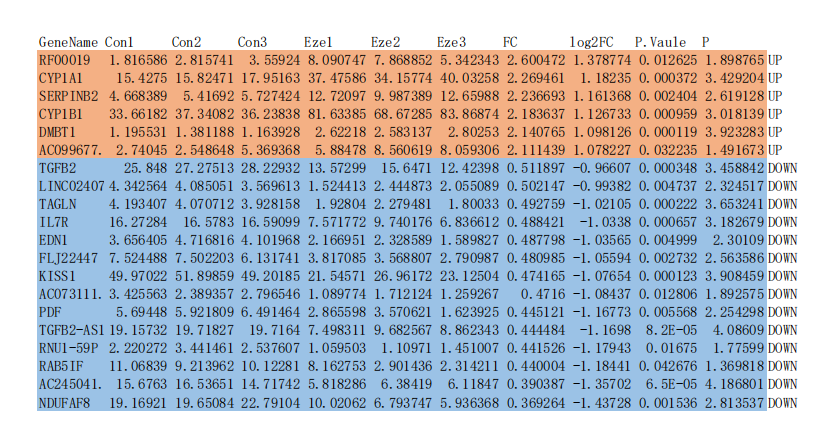


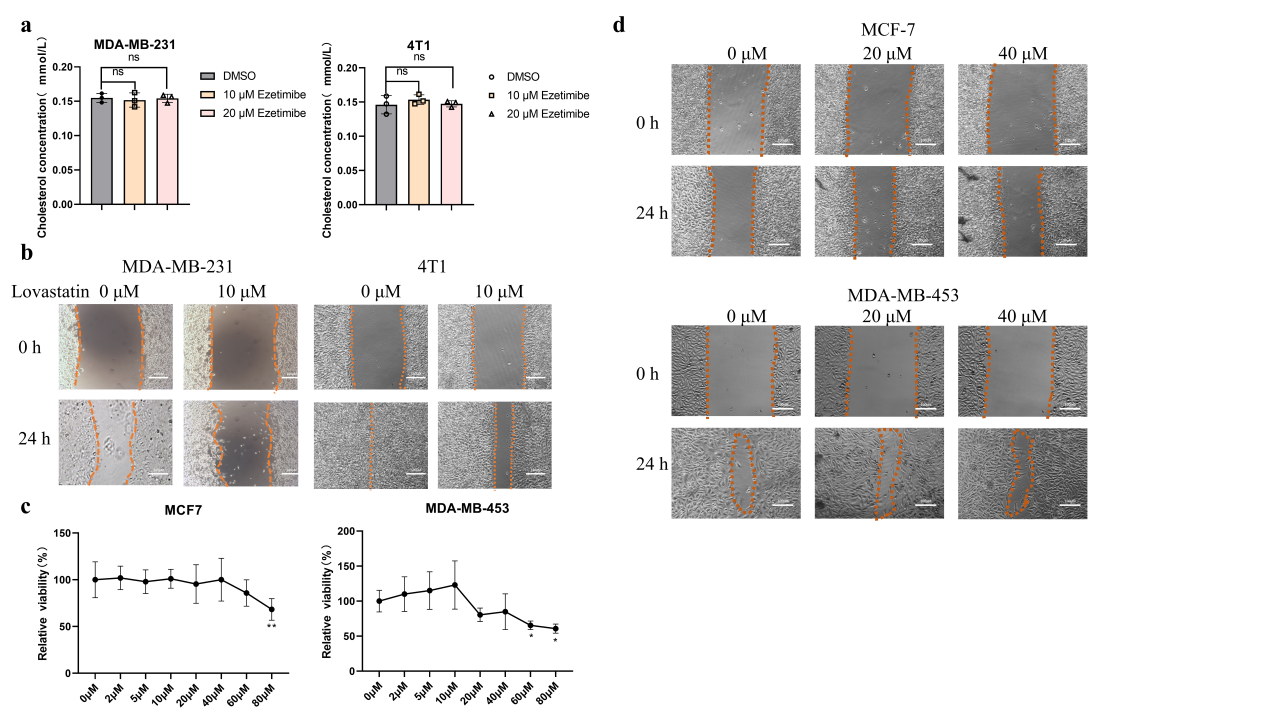


**Fig S2.** **The effect of ezetimibe on cholesterol absorption in TNBC and its effect on cell viability and migration in luminal A (MCF7) and HER2-enriched (MDA-MB-453) breast cancer cells.** (a) MDA-MB-231 and 4T1 cells were treated with 10 or 20 µM ezetimibe for 24 hours, afterward, cell supernatants were collected, and the cholesterol content was measured. The results shown in the figures are representative of three independent experiments, and the results are expressed as the mean ± standard deviation (mean ± SD). (b) MDA-MB-231 and 4T1 cells were treated with 10 µM lovastatin for 24 hours, after which a scratch healing assay was used to determine the cell healing rate. Cell images at 0 h and 24 h were taken under a light microscope at a magnification of 40× in three random fields for each experimental setting. (c) MCF7 and MDA-MB-453 cells were treated with different concentrations of ezetimibe (0, 1, 5, 10, 20, 40, 60, 80 µM) for 48 hours, after which cell viability was assessed using the CCK-8 assay. The experiment was repeated three times independently with six complex wells per group, and the results are shown as the mean ± standard deviation (mean ± SD).* P < 0.05, * * P < 0.01.(d) MCF7 and MDA-MB-453 cells were treated with 10, 40 µM ezetimibe for 24 hours, after which a scratch healing assay was used to determine the cell healing rate. Cell images at 0 h and 24 h were taken under a light microscope at a magnification of 40× in three random fields for each experimental setting.


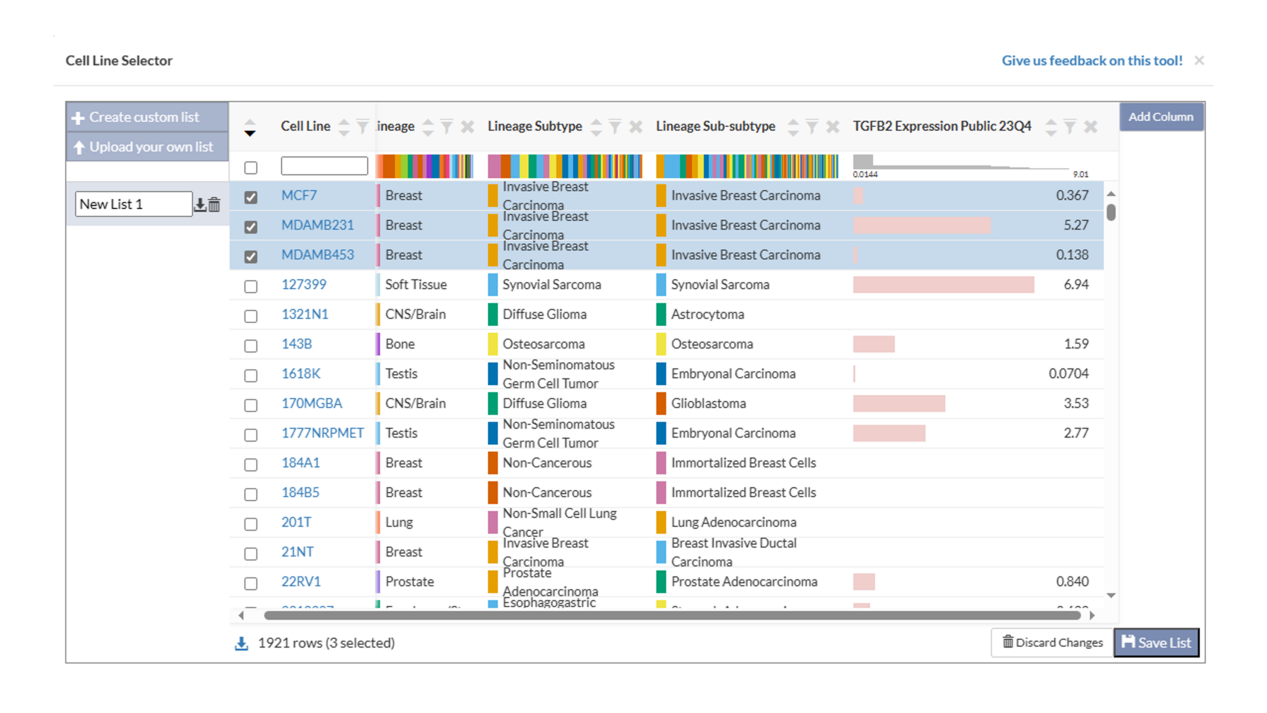


**Fig S3. The Cancer Cell Line Encyclopedia (CCLE) database was used to analyze the mRNA expression of TGFB2 in the MCF7, MDA-MB-453, and MDA-MB-231 cell lines.**
